# Evaluation of Selective Survival and Sex/Gender Differences in Dementia Incidence Using a Simulation Model

**DOI:** 10.1001/jamanetworkopen.2021.1001

**Published:** 2021-03-09

**Authors:** Crystal Shaw, Eleanor Hayes-Larson, M. Maria Glymour, Carole Dufouil, Timothy J. Hohman, Rachel A. Whitmer, Lindsay C. Kobayashi, Ron Brookmeyer, Elizabeth Rose Mayeda

**Affiliations:** 1 Fielding School of Public Health, Department of Epidemiology, University of California, Los Angeles; 2 Fielding School of Public Health, Department of Biostatistics, University of California, Los Angeles; 3 Department of Epidemiology and Biostatistics, University of California, San Francisco; 4 Centre Inserm U1219, d’Epidémiologie et de Développement, Bordeaux School of Public Health, Institut de Santé Publique Université de Bordeaux, Bordeaux, France; 5 Pole de sante publique, Centre Hospitalier Universitaire de Bordeaux, Bordeaux, France; 6 Vanderbilt Memory and Alzheimer’s Center, Vanderbilt University Medical Center, Nashville, Tennessee; 7 Vanderbilt Genetics Institute, Vanderbilt University Medical Center, Nashville, Tennessee; 8 Alzheimer’s Disease Research Center, University of California, Davis; 9 Department of Public Health Sciences, University of California, Davis; 10 Department of Epidemiology, University of Michigan School of Public Health, Ann Arbor

## Abstract

**Importance:**

Dementia research is susceptible to bias arising from selective survival, a process that results in individuals with certain characteristics disproportionately surviving to old age. Spurious associations between risk factors and dementia may be induced when factors associated with longer survival also influence dementia incidence.

**Objective:**

To assess the role of selective survival in explaining reported sex/gender differences in dementia incidence.

**Design, Setting, and Participants:**

This decision analytical model used a simulated cohort of US participants aged 50 years and without dementia at baseline followed up for incident dementia through age 95 years. Selective survival was induced by a selection characteristic (eg, childhood social disadvantage or Alzheimer genetic risk) that influenced both mortality and dementia incidence at varying magnitudes. Data analysis was performed from April 2018 to May 2020.

**Exposure:**

Sex/gender, conceptualized as the combination of biological sex and social consequences of gender.

**Main Outcomes and Measures:**

Dementia incidence rate ratios (IRRs) for women compared with men. In all simulations, it was assumed that there would be no true effect of sex/gender on dementia incidence; all observed sex/gender differences were due to selective survival.

**Results:**

At baseline, the simulation included 100 000 participants aged 50 years (51 000 [51%] women, mirroring the 1919-1921 US birth cohort of non-Latino White individuals at age 50 years); distributions of the selection characteristic were standard normal (mean [SD], 0.0 [1.0]). Observed sex/gender differences in dementia incidence in individuals aged 85 years or older ranged from insignificant (IRR, 1.00; 95% CI, 0.91-1.11) to consistent with sex/gender differences (20% higher risk for women [IRR, 1.20; 95% CI, 1.08-1.32]) reported in an extant study. Simulations in which bias was large enough to explain prior findings required moderate to large differential effects of selective survival (eg, hazard ratio for selection characteristic on mortality at least 2.0 among men, no effect among women).

**Conclusions and Relevance:**

These results suggest that selective survival may contribute to observed sex/gender differences in dementia incidence but do not preclude potential contributions of sex/gender-specific mechanisms. Further research on plausibility of selection characteristics with outcomes of the magnitude required for selective survival to explain sex/gender differences in dementia incidence and sex/gender-specific mechanisms represent an opportunity to understand prevention and treatment of dementia.

## Key Points

**Question:**

Can selective survival plausibly explain reported sex/gender differences in dementia incidence?

**Findings:**

In this decision analytical model of 100 000 simulated adults aged 50 years and without dementia at baseline, sex/gender differences in dementia incidence consistent with literature (ie, 15%-20% elevated risk for women aged ≥85 years) were only observed in the presence of moderate to strong effects of selective survival characteristics that differed by sex/gender.

**Meaning:**

These findings suggest that selective survival may contribute to sex/gender differences in dementia incidence but do not preclude the potential for additional contributions from biological mechanisms.

## Teaser

This decision analytical model assesses the potential role of selective survival in sex/gender differences in dementia incidence.

## Introduction

Most people living with dementia are women.^1^ Excess dementia burden among women compared with men could be explained by women’s longer life expectancy, higher age-specific dementia incidence rates among women, or selective survival bias.^2^ Some studies report higher age-specific dementia incidence in women compared with men at older ages (ie, ages ≥85 years),^3,4,5,6,7,8,9^ while other studies have not reported differences.^10,11,12^ Mechanisms triggered by either biological sex or social consequences of gender could contribute to the difference.^10,13,14,15^ Selective survival bias can occur if men (or women) with specific characteristics disproportionately survive to old age and those same characteristics are associated with dementia risk.^16,17,18,19^ Subsequently, measures of association among survivors may not represent causal effects and can be exaggerated, attenuated, or reversed compared with the truth.^16^ The magnitude of bias is driven by the strength of association between exposure of interest and mortality, strength of association between dementia and mortality, and cumulative mortality.^20,21^

If true effects of sex/gender on dementia incidence exist—differences not attributable to selective survival bias—identifying mechanisms for this inequality may inform development of effective strategies to prevent or treat dementia in both men and women.^22,23^ This has motivated research focused on sex/gender differences in dementia incidence, pathological processes associated with dementia,^24,25,26^ and resilience to dementia-related pathological processes^27,28^; but there are no formal evaluations of the role of selective survival bias.^10,11,14^

Simulation studies are useful for quantifying potential magnitudes of selective survival bias.^18,19^ Motivated by prior evidence documenting elevated dementia incidence among older women compared with men,^3,4,5,6,7^ we developed a decision analytical model to simulate and quantify the extent to which selective survival may contribute to estimates of sex/gender differences in dementia incidence.

## Methods

This decision analytical model included simulations that did not include individual-level data; thus, institutional review board review was not required per institutional policy at the University of California, Los Angeles. Reporting of this study follows the relevant noncost aspects of the Consolidated Health Economic Evaluation Reporting Standards (CHEERS) reporting guideline for decision analytical model studies.

### Simulated Cohort Study

We simulated a cohort of 100 000 participants aged 50 years without dementia at baseline (51 000 [51%] women, mirroring the 1919-1921 US birth cohort of non-Latino White individuals at age 50 years^29^). The simulated cohort was followed for incident dementia through age 95 years (45-year follow-up). We chose a sample size of 100 000 to balance computational feasibility while maximizing the number of survivors at the oldest ages. We considered several causal scenarios reflecting selective survival that might explain sex/gender differences in dementia incidence, with input parameters guided by real-world data. We use the term *sex/gender* to recognize that either biological or social mechanisms may contribute to observed excess dementia among women; this distinction would not affect findings from simulations.

### Causal Scenarios

**Figure 1.**
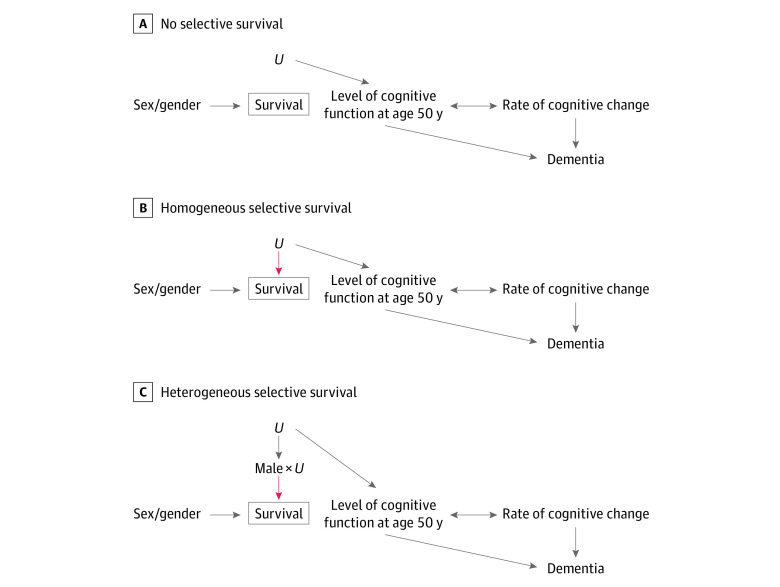
Causal Scenarios Under Investigation In all scenarios, sex/gender affected survival while *U* (selection characteristic) influenced level of cognitive function at age 50 years (the age at baseline in all simulations) and subsequent dementia incidence. In the no selective survival scenario (A), *U* had no effect on survival. In the homogeneous selective survival scenario (B), *U* affected survival for both men and women. In the heterogeneous selective survival scenario (C), *U* affected survival for men only (interaction effect between *U* and sex/gender). The red arrows in B and C highlight *U*’s effect on survival, which induces the selective survival process. Although it is unconventional to show interaction terms in directed acyclic graphs, the pseudo–directed acyclic graph in C explicitly shows the sex/gender-*U* interaction term for clarity.

We investigated 3 causal scenarios (Figure 1). Arrows represent causal relationships specified for each simulation scenario. Associations induced by the causal structures (not specified in the data-generating process directly), are not marked in the diagrams. In all scenarios, the level of cognitive function at age 50 years and rate of cognitive change after age 50 years influenced dementia incidence. The double-headed arrow between cognitive function at age 50 years and rate of cognitive change indicates that these variables were simulated as correlated, reflecting a potential common cause or causal path in either direction. To quantify the extent to which selective survival may explain higher dementia incidence in women vs men at older ages, we generated data under the sharp null hypothesis of no sex/gender effect on dementia incidence for individuals in the population. Thus, in our simulations, observed sex/gender differences in dementia incidence reflect selective survival bias.

To represent the selective survival process, we included a variable *U* (normally distributed with mean [SD], 0.0 [1.0]), that represented the set of characteristics (eg, childhood social disadvantage or Alzheimer genetic risk), that influenced cognitive function at age 50 years and, in causal scenarios with possible selective survival bias, survival. Larger *U* values were harmful to survival and cognitive function. We specified a causal structure such that at age 50 years, *U* was unrelated to sex/gender. Because life expectancy is longer for women than men,^29^ we expected that as the cohort aged, individuals with values of *U* protective against dementia (smaller values) would be overrepresented among surviving men. As a result, we expected lower dementia incidence among surviving men compared with surviving women at older ages.

To ensure simulations worked as expected, we calibrated them using a scenario in which no bias was anticipated. In the no selective survival scenario, *U* influenced level of cognitive function at age 50 years, but did not influence survival (Figure 1A). All other simulated scenarios could potentially give rise to selective survival bias. In the homogeneous selective survival scenario, *U* decreased survival for men and women (Figure 1B). In the heterogeneous selective survival scenario, *U* decreased survival in men only (interaction between *U* and sex/gender, such that *U* influenced mortality for men only) (Figure 1C). We did not simulate a scenario in which *U* influenced mortality for women only because this would not produce elevated dementia incidence in women and therefore could not explain observed higher dementia incidence among women.

### Data-Generating Process

In all scenarios, we generated data specifying no true effect of sex/gender on dementia. Age-specific mortality and dementia incidence rates in simulations were calibrated to real-world data. Mortality was calibrated to US lifetable data for non-Latino White individuals born 1919 to 1921^29^; this birth cohort is representative of the cohort used to calibrate dementia incidence in our simulations and is the most recent birth cohort with published mortality data through age 95.^7^ Lifetable data were taken in 5-year age bands for ages 50 to 95 years. To generate sex/gender-specific survival distributions calibrated to match the 1919 to 1921 US birth cohort of non-Latino White individuals, we used mortality hazard models, allowing the baseline mortality hazard and effect of sex/gender on mortality to vary across 5-year age bands (eAppendix in the Supplement).

Dementia incidence was calibrated to a 2005 study by Tom et al^7^ that reported age- and sex/gender-specific dementia incidence rates from the Adult Changes in Thought (ACT) study, a contemporary (ie, aged ≥65 years in 1994-2010) US population.^30^ Because dementia incidence rates vary by study and tend to be imprecisely estimated at older ages owing to small sample sizes (eg, 120 to 250 adults aged ≥85 years),^3,4,7,31,32,33,34,35,36^ reported dementia incidence rates were used as a guide rather than a strict calibration criterion. Incident dementia in simulations was conceptualized as reflecting the culmination of a continuous process of cognitive decline; we generated person-specific cognitive trajectories from age 50 years using a quadratic growth curve with random intercepts, random linear slopes, and random quadratic slopes. Individuals could develop dementia in 2 ways: either their cognitive function declined below an age-constant dementia cutoff or they experienced a random shock event (eg, a serious stroke) that triggered dementia onset (eAppendix in the Supplement). Mean cognitive trajectories and samples of individual trajectories are presented in eAppendix in the Supplement.

We varied effects of *U* on mortality and cognitive function within investigated causal scenarios. We used log(2.0) as the age-constant effect of U on log mortality hazard as a moderate effect size and log(3.5) as a large effect size. For the moderate effect, a 1-SD increase in *U* increased the mortality hazard 2-fold; for the large effect, a 1-SD increase in *U* increased mortality hazard 3.5-fold. In the heterogeneous selective survival scenario, *U* affected mortality hazard for men only. Moderate effects of U on cognition were set to −0.1 (moderate homogeneous selective survival scenario and moderate heterogeneous selective survival scenario) and large effects to −0.5 (large homogeneous selective survival scenario and large heterogeneous selective survival scenario). Thus, 1-SD higher *U* value was associated with 0.1 SD lower cognitive function at age 50 years in moderate scenarios and with a 0.5 SD lower cognitive function in scenarios with large effects.

### Statistical Analysis

We quantified bias in sex/gender differences in age-specific dementia incidence rates induced by selective survival. The ACT study reported by Tom et al^7^ and several other studies^3,4,5,6^ reported sex/gender differences in dementia (estimated incidence rate ratio [IRR] for women vs men, >1.00) for participants aged 85 years or older. In the ACT study,^7^ the estimated dementia IRR for women vs men was 1.27 (95% CI, 0.96-1.69) for individuals aged 85 to 89 years and estimated dementia IRR for women vs men was 1.10 (95% CI, 0.74-1.63) for individuals aged 90 to 94 years (eAppendix in the Supplement).

For each sample across 1000 iterations of sample generation, we calculated the age band–specific dementia IRR for women vs men. Sex/gender-specific IRRs were calculated by summing incident dementia cases within an age band and dividing by total person-years at risk within the age band. We quantified the magnitude of bias induced by selective survival for each scenario by comparing the exponentiated mean of the age-band specific log(estimated IRR for women vs men) with the true effect of sex/gender on dementia incidence in simulated data (IRR for women vs men = 1.00). By comparing estimated sex/gender differences in dementia incidence with simulation truth (no effect), we quantified the extent to which selective survival explained observed differences in simulations.

Simulations were carried out using R statistical software version 3.6.2 (R Project for Statistical Computing). *P* values were 2-sided, and statistical significance was set at *P* < .05. Data analysis was performed from April 2018 to May 2020.

## Results

At baseline, the cohort included 100 000 simulated participants aged 50 years, with 51 000 (51%) women, and selection characteristic *U* was standard normal (mean [SD], 0.0 [1.0]). In all simulation scenarios, median (interquartile range) survival time from age 50 years was 22.6 (14.3-30.2) years for men and 23.6 (15.3-31.0) years for women. Mean (SD) cumulative incidence of mortality by age 95 years was 99.1% (<0.1%), consistent with US life tables for the selected birth cohort.^29^ In all simulation scenarios, dementia incidence rates for men (used as the calibration reference) approximated age-specific dementia incidence rates in the ACT study^7^ (calibration sample) (eAppendix in the Supplement).

**Figure 2.**
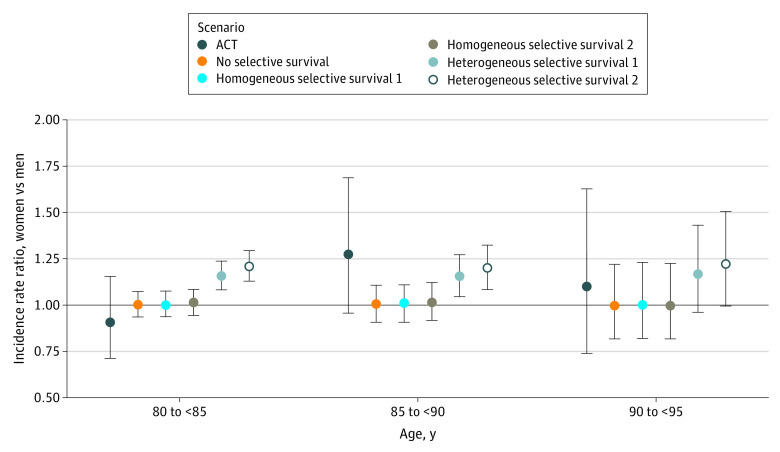
Mean Estimated Dementia Incidence Rate Ratio (IRR) for Women vs Men for Individuals Aged 80 Years and Older Across 1000 Simulated Cohorts for All Scenarios Compared With the Adult Changes in Thought Study Data were acquired from the Adult Changes in Thought study as reported by Tom et al.^7^ The mean estimated IRR was calculated across 1000 simulated cohorts as *e*^mean(log[estimated IRR women vs men])^. Homogeneous selective survival 1 and heterogeneous selective survival 1 indicate scenarios with moderate effect sizes for *U*; homogeneous selective survival 2 and heterogeneous selective survival 2 indicate scenarios with large effect sizes for *U*. Error bars indicate 95% CIs for each estimate; black line, estimated dementia IRR of 1.0 (ie, the null value).

**Figure 3.**
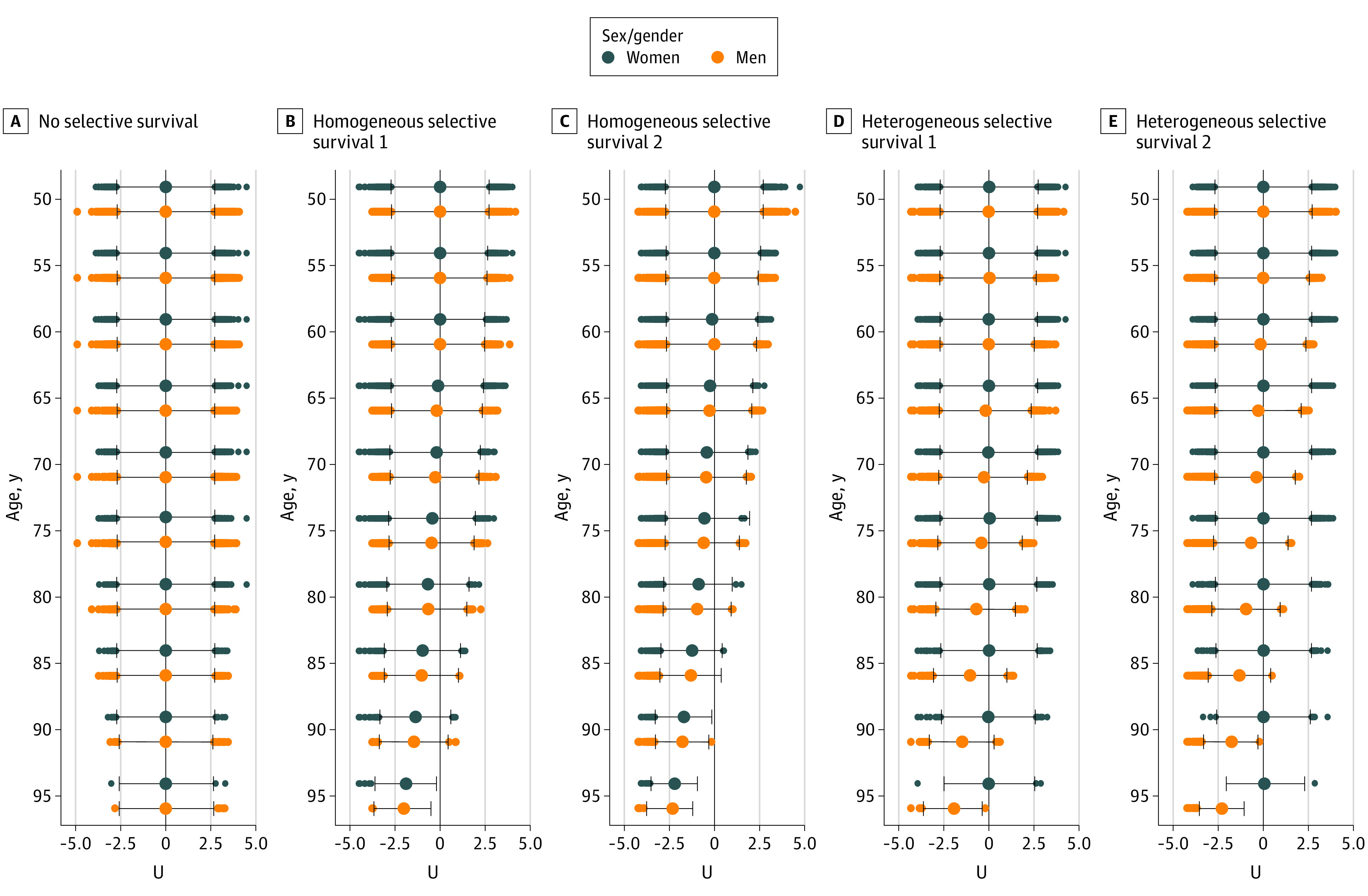
Box Plots of Selection Characteristic, *U*, by Age and Sex/Gender for All Simulation Scenarios Distributions presented are for 1 cohort of 100 000 individuals. The vertical black line at 0.0 denotes the mean value of *U* at baseline (age 50 years). Homogeneous selective survival 1 and heterogeneous selective survival 1 indicate scenarios with moderate effect sizes for *U*; homogeneous selective survival 2 and heterogeneous selective survival 2 indicate scenarios with large effect sizes for *U*.

We used the no selective survival scenario to check the simulation model. In this scenario, the mean estimated dementia IRR for women vs men was unbiased in all age bands, as expected. Figure 2 shows that in the no selective survival scenario, mean estimated dementia IRR for women vs men was 1.00 (95% CI, 0.91-1.11) for ages 85 to 89 years and 1.00 (95% CI, 0.82-1.22) for ages 90 to 94 years. Figure 3 shows that *U* distributions remained standard normal in this scenario through the end of follow-up.

In the moderate homogeneous selective survival scenario, mean estimated dementia IRR for women vs men was 1.00 (95% CI, 0.91-1.11) for ages 85 to 89 years and 1.01 (95% CI, 0.82-1.23) for ages 90 to 94 years. For the same causal structure, but with large input parameters (the large homogeneous selective survival scenario), mean estimated dementia IRR for women vs men was 1.02 (95% CI, 0.92-1.12) for ages 85 to 89 years and 1.00 (95% CI, 0.82-1.23) for ages 90 to 94 years. In both scenarios, *U* distributions for both men and women shifted to smaller (ie, more protective) values as the cohort aged (ie, survivors became more selected for protective *U* values over time); this shift was more pronounced in the large homogeneous selective survival scenario. Precise numbers varied across simulations owing to chance, but *U* distributions in these simulations differed little for men and women. For example, in 1 simulated cohort for the moderate homogeneous selective survival scenario, mean (SD) *U* at age 95 years (ie, end of follow-up) was −2.05 (0.62) for men and −1.94 (0.72) for women; in the large homogeneous selective survival scenario, mean (SD) *U* at age 95 years was −2.35 (0.55) for men and −2.24 (0.54) for women (Figure 3). Thus, the populations of men and women survivors at age 95 years were similarly enriched in factors, represented by *U*, that protected against mortality and dementia. This is consistent with our result of no sex/gender differences in dementia incidence in this simulation scenario.

The heterogeneous selective survival scenarios produced estimates that were consistent with estimates from the ACT study^7^ at older ages. In the moderate heterogeneous selective survival scenario, mean estimated dementia IRR for women vs men was 1.15 (95% CI, 1.05-1.27) for ages 85 to 89 years and 1.17 (95% CI, 0.96-1.43) for ages 90 to 94 years. For the large heterogeneous selective survival scenario, mean estimated dementia IRR for women vs men was 1.20 (95% CI, 1.08-1.32) for ages 85 to 89 years and 1.22 (95% CI, 1.00-1.51) for ages 90 to 94 years. In both heterogeneous selective survival scenarios, the selective survival process manifested in differences in *U* distributions for surviving men and women: surviving men were more selected for protective values of *U* than surviving women. Differences in *U* distributions for surviving men and women were more pronounced in the large heterogeneous selective survival scenario. For example, in 1 simulated cohort of the moderate heterogeneous selective survival scenario, mean *U* at age 95 years was −1.94 (0.66) for men and −0.02 (0.99) for women; for the large heterogeneous selective survival scenario, mean *U* at age 95 years was −2.32 (0.54) for men and 0.08 (0.92) for women.

## Discussion

This decision analytical model assessed the role of selective survival in explaining sex/gender differences in dementia incidence by simulating cohorts under varying causal scenarios. In all simulations, we generated data specifying no true effect of sex/gender on dementia incidence; thus, any sex/gender differences in estimated dementia incidence in simulations reflected selective survival bias. In homogeneous selective survival scenarios, we observed little to no selective survival bias (ie, mean estimated dementia IRR for women vs men close to 1.00). However, the heterogeneous selective survival scenarios produced enough selective survival bias to generate estimates consistent with prior results from the ACT study reported by Tom et al,^7^ showing 10% to 27% higher dementia incidence among women at age 85 years and older (Figure 2); the large heterogeneous selective survival scenario produced estimates closest to those reported in the ACT study,^7^ with 20% to 22% higher dementia incidence in women compared with men at the oldest ages.

In our simulations, a differential selection process (heterogeneous selective survival scenario) was necessary to produce notable sex/gender differences in dementia incidence. This suggests that research on factors that are associated with dementia risk and are differentially associated with survival for men vs women is necessary to gain a comprehensive understanding of the role of selective survival in sex/gender differences in dementia incidence. In our simulations, *U* represented a set of selection characteristics. To produce sufficient selective survival bias to fully explain sex/gender differences in dementia incidence, our simulations suggest the necessity of selection characteristics with moderate to large effect size: for every 1-SD increase, the characteristics would have to increase mortality approximately 2-fold for men only and reduce cognitive function by 0.1 SD.

One example of a factor with an effect size of this magnitude is cardiovascular disease (CVD), which kills men at 2- to 6-fold the rate at which it kills women^37,38^ and shares risk factors with dementia.^39^ CVD is differentially distributed among men and women; thus, CVD is an example of a *U* that is influenced by sex/gender. In our simulations, sex/gender did not influence *U*, but prior simulation work demonstrates that causal structures in which *U* mediates the effect of sex/gender on dementia would produce results consistent with those from structures such as ours, in which *U* is not on the causal pathway of interest.^19^ Future simulations could explore the complex associations between sex/gender, CVD, mortality, and dementia.

Most work on sex/gender differences in dementia focuses on sex/gender differences in susceptibility to dementia-related pathological processes.^24,25,26,28^ A 2020 review by Ferretti et al^13^ outlines hypothesized sex/gender-specific risk factors for Alzheimer pathological processes (eg cardiovascular risk factors, depression, APOE*E4 genotype, and historically limited access to high-level education and societal leadership roles for women) and pathophysiological mechanisms for these differences (immune system and mitochondrial cascade). Some sex/gender differences in associations of risk factors with dementia or dementia-related pathological processes, such as APOE*E4,^40^ are generally accepted. Other active avenues of research include possible sex/gender-specific susceptibility to amyloid-β and τ pathological mechanisms.^24,25,26,28^ However, our findings suggest that a more comprehensive picture of possible mechanisms that drive sex/gender differences in dementia incidence should include the possibility that selective survival accounts for at least some of the disparity. Because the role of selective survival bias depends on existence of dementia risk factors that have differential effects on survival for women vs men, identifying such risk factors is essential for understanding sex/gender differences in dementia.

### Limitations

This study has some limitations, and there are simplifying assumptions in our simulations. For simplicity in the heterogeneous selective survival scenario, we specified that *U* only affected survival for men. We anticipate that any scenarios with heterogenous selection (eg, *U* affects women’s survival but has larger effects on men’s survival) would yield similar results. Second, sex/gender did not influence *U* in simulations; including an effect of sex/gender on *U* (eg, cardiometabolic health) is an alternative causal structure in which sex/gender has a true causal effect on dementia (indirectly through *U*). In this study, we were interested in whether higher dementia incidence rates among women vs men could be entirely explained by selective survival. Prior simulation studies have suggested that results would be consistent whether or not *U* was on the causal pathway of interest.^19^

Additionally, our simulations were constrained by available calibration data. For example, we calibrated simulations to all-cause dementia. Future simulation work could evaluate roles of selective survival and selective study participation on estimates of sex/gender differences in dementia subtypes, pathological mechanisms associated with dementia, and effects of risk factors on dementia and pathological mechanisms associated with dementia. There is limited research on sex/gender differences in dementia in racially/ethnically diverse populations; however, a 2019 study by Avila et al^41^ suggested that sex/gender differences in cognitive performance may vary by race/ethnicity. Simulations in this study were calibrated to all-cause dementia incidence from the ACT study,^7^ a sample comprising predominantly non-Latino White participants. Our findings may not be generalizable to other populations. Differences in cumulative mortality (overall and by sex/gender) across populations could influence the potential role of selective survival on sex/gender differences in dementia incidence.^18,21^

## Conclusions

The results of this decision analytical model pertain to the role of selective survival bias in understanding sex/gender differences in dementia incidence, but lessons from this example can be applied to dementia research more broadly. Sources and potential impacts of selective survival bias are study-specific; for example, racial/ethnic differences in survival^18^ or differential survival among persons with hypertension or other comorbidities,^19,42^ may represent other sources of selective survival in dementia research. Researchers might anticipate and mitigate such bias by considering relevant selection characteristics in the design phase, collecting data on those characteristics, and adjusting for them in analyses.^16^ Because it is often not possible to measure all relevant selection characteristics, simulations can be used to assess the extent to which unmeasured selection characteristics could bias study results. Our results suggest that selective survival may contribute to, and under some more extreme scenarios, may fully explain sex/gender differences in dementia incidence, but our results do not preclude the potential contribution of sex/gender-specific mechanisms for dementia. Importantly, if sex/gender differences in dementia incidence are small after accounting for selective survival, this does not diminish the value of researching potential sex/gender-specific mechanisms. Since most people living with dementia are women, it is critical to determine the role of sex/gender in dementia risk. Further research on important determinants of dementia that have differential effects on survival for women vs men and sex/gender-specific mechanisms represents an opportunity to identify potential strategies to prevent and treat Alzheimer and related dementias.

## Notes

Supplement. **eAppendix.** Supplemental Methods **eReferences.**
