# Supplementary material for: Evaluation of Selective Survival and Sex/Gender Differences in Dementia Incidence Using a Simulation Model
Source: JAMA Netw Open. 2021 Mar 9;4(3):e211001. doi: 10.1001/jamanetworkopen.2021.1001 (PMC7944377; doi:10.1001/jamanetworkopen.2021.1001)
Supplement: Supplement. — eAppendix. Supplemental Methods eReferences. [file jamanetwopen-e211001-s001.pdf]

Supplemental Online Content

Shaw C, Hayes-Larson E, Glymour MM, et al. Evaluation of selective survival and sex/gender differences in dementia incidence using a simulation model. *JAMA Netw Open*. 2021;4(3):e211001. doi:10.1001/jamanetworkopen.2021.1001

eAppendix. Supplemental Methods
eReferences.

This supplemental material has been provided by the authors to give readers additional information about their work.

© 2021 Shaw C et al. *JAMA Network Open.*

eAppendix. Supplemental Methods

Additional Data-Generating Process Details

Code for all calibrations and analyses can be found on the Mayeda Research Group GitHub: (https://github.com/Mayeda-Research-Group/Simulation-Study-Sex-Dementia).

Survival Function

To match survival rates in our simulations to US lifetable data, we calibrated the baseline mortality rate in men and the effect female sex/gender on mortality such that sex/gender-specific cumulative survival from age 50 matched lifetables. Here, we discuss the mortality hazard model and details of the calibration process.

For each individual in each five-year age band, [50-55), [55-60), …, [90-95), time to death was generated as a random variable drawn from an exponential survival distribution. In all simulation scenarios, we generated survival times for individual i in age band j as a random variable drawn from an exponential survival distribution based on the hazard function in Equation A.1. We assumed constant baseline mortality hazard λ_j within each age band j:

$$h_{death_{ij}}(t) = \lambda_j exp\{\gamma_{1j} female_i + \gamma_2 U_i + \gamma_3 U_i \times male_i\}, \quad \text{(A.1)}$$

where $U_i \sim N(0, 1)$ and t is the time from baseline study visit. Note that participants' ages are not included in the model because everyone is the same age at baseline in this simulation, thus there is no age effect to account for beyond the differences in λ_j between age bands. We calibrated survival in our simulations to the US 1919-1921 non-Latino white birth cohort. Calibrating survival in our simulations to US lifetable data involved choosing appropriate parameters for the hazard function so that the conditional probability of survival (survival to age $x + 5$ conditional on survival to age x) for men and hazard ratios for mortality (women versus men) in our simulation closely matched those calculated in the lifetables. For each scenario, we fixed the effect of U on log hazard of death. The values chosen for these effects in each scenario are reported in **eTable 1**.

We used R's optim function to solve for values of λ_j, the baseline mortality hazard for men in each 5-year age band, so that conditional probabilities of survival for men in each band closely matched those from US lifetables for each age band. We used the resulting optimized values of λ_j in the model and R's optim function to solve for values of γ_{1j}, the effect of female sex/gender on log hazard of death, so that mortality hazard ratios (women versus men) closely matched those calculated from US lifetables. **eFigure 1** illustrates the success of our calibration for (a) survival probabilities and (b) mortality hazard ratios.

Cognitive Trajectories and Dementia

Calibrating dementia incidence rates in our simulation to real data involved choosing appropriate parameters in the model for cognitive trajectories, choosing an age-constant dementia cut-point (i.e., a threshold for cognitive function below which an individual would be classified as having dementia), and choosing a value for the constant rate of "random shock" dementia that together would produce reasonable cognitive trajectories (not too steep) and reasonable dementia incidence rates (reflective of real data). Here, we discuss the details for this calibration process, the resulting parameters, and the success of the calibration to dementia incidence rates for men (used as the reference group) reported in the Adult Changes in Thought study, which reported contemporary (1994-2010) age- and sex/gender-specific dementia incidence rates in a US population.[1,2]

In all simulation scenarios, individuals could develop dementia in two ways: (1) their cognitive function fell below an age-constant dementia cut-point or (2) they experienced a "random shock" event (e.g. a serious stroke) that gave them dementia immediately. We used an age-constant rate for the "random shock" events. We generated person-specific cognitive trajectories from age 50 using a quadratic growth curve for cognitive decline with a random intercept, random linear slope, and random quadratic slope. Cognitive function C_i for individual i at time t, where t is the number of years from baseline, was determined by the quadratic mixed effects model defined in Equation A.2.

$$C_i(t) = \beta_{00} + \zeta_{0i} + \beta_{01} U_i + \varepsilon_i + (\beta_{10} + \zeta_{1i})t + (\beta_{20} + \zeta_{2i})t^2, \quad \text{(A.2)}$$

where $U_i \sim N(0, 1)$. In this model, $\varepsilon_i \sim N(0, \sigma_\varepsilon^2)$ represents unexplained variation in C_i and is independent of all other random effects in the model. We centered the random effects (ζ_i terms) at 0 and specified the following covariance structure for ζ_0, ζ_1, ζ_2 (suggesting that individual random intercepts were slightly negatively associated with individual random coefficients for linear decline, but both were independent of the quadratic term):

© 2021 Shaw C et al. *JAMA Network Open.*

$$Z = \begin{bmatrix} 5.0 \times 10^{-2} & -9.0 \times 10^{-5} & 0 \\ -9.0 \times 10^{-5} & 1.0 \times 10^{-3} & 0 \\ 0 & 0 & 9.0 \times 10^{-6} \end{bmatrix}.$$

To develop dementia due to cognitive decline, an individual's cognitive trajectory had to fall below an age-constant cut point for dementia. Parameters for the C_i model were determined simultaneously with this age-constant dementia cut point so that together, C_i trajectories would represent reasonable rates of decline and reasonable dementia incidence rates (reflective of reported rates). The age-constant cut point used across the simulation scenarios was -6.5. This cut-point was standardized to the distribution of cognitive function for 50-year-olds in our simulation which was roughly standard normal (mean = 0, SD = 1.0). Thus, an individual developed dementia due to cognitive decline when their cognitive function fell below 6.5 standard deviations below 0 in the distribution of cognitive function for 50-year-olds, regardless of their age. Because this cut-point is so extreme for individuals at younger ages, there were no incident dementia cases in our simulation until the [65, 70) age-band.

To ensure that individuals' cognitive trajectories declined continuously, we guaranteed a negative quadratic coefficient in our models for C_i (Equation A.2) by taking the negative absolute value of the value drawn for ζ_{2i} (the random quadratic slope) (i.e., by drawing ζ_{2i} from a half-normal distribution). The cognitive intercept was set to 0 in all scenarios to obtain approximately standard normal distributions of baseline cognitive function. Values for the linear and quadratic coefficients in the model for C_i in each simulation scenario were obtained through hand calibration (plugging in values and testing resulting dementia incidence rates) so that dementia incidence rates for men matched those reported in the ACT study. The parameters used for the C_i model in each simulation scenario are presented in **eTable 2**. Average cognitive trajectories for men and women and samples of individual trajectories in each simulation scenario are presented in **eFigure 2**. We determined the rate of "random shock" dementia from incidence rates for men in the youngest age bands of the ACT study [65, 70). We set the rate of "random shock" dementia in our simulations to 7/1000 person-years for every age band, based on the dementia incidence rate reported for men in the youngest age band, [65, 70).

There was no interval censoring in this simulation study. For those diagnosed with dementia due to cognitive decline in a specified time interval, we solved for the time within that interval that the individual's cognitive trajectory fell below the age-constant dementia cut point. For an individual who developed dementia based on a "random shock" event in a specified time interval, their time to dementia was determined by first drawing a random variable uniformly from the interval (0, 5) (corresponding to the time between "study visits"). This random draw was then added to the years from baseline visit at the start of the time interval.

Dementia incidence rates reported in the ACT study were used as a guide rather than a strict calibration criterion because of the likely chance variation in ACT results across age bands (reflected by wide confidence intervals). To ensure the validity of our simulation results, we verified that each simulation scenario was as well-calibrated to the ACT study data as the other simulation scenarios. The consistency of our calibration is shows in **eFigure 3**, which depicts the dementia incidence rates for men in each of our simulation scenarios compared to the rates reported for men in the ACT study. The average dementia $\widehat{IRR}$ in all our simulations compared to those reported in the ACT study are presented in **eTable 3**.

© 2021 Shaw C et al. *JAMA Network Open.*

eReferences

1. Tom SE, Hubbard RA, Crane PK, et al. Characterization of dementia and Alzheimer's disease in an older population: Updated incidence and life expectancy with and without dementia. *Am J Public Health*. 2015;105(2):408-413. doi:10.2105/AJPH.2014.301935
2. Kukull WA, Higdon R, Bowen JD, et al. Dementia and Alzheimer disease incidence: A prospective cohort study. *Arch Neurol*. 2002;59(11):1737-1746. doi:10.1001/archneur.59.11.1737

© 2021 Shaw C et al. *JAMA Network Open.*

eTable 1. Parameter Inputs for Mortality Hazard Model in Equation A.1 **($h_{death_{ij}}(t) = \lambda_j exp\{\gamma_{1j} female_i + \gamma_2 U_i + \gamma_3 U_i \times male_i\}$) for all simulation scenarios.***

Parameter	No Selective Survival	HOM1	HOM2	HET1	HET2
γ_2	0	log(2)	log(3.5)	0	0
γ_3	0	0	0	log(2)	log(3.5)

*No Selective Survival: Scenario without selective survival; HOM1: Homogeneous Selective Survival scenario with moderate input parameters; HOM2: Homogeneous Selective Survival scenario with large input parameters; HET1: Heterogeneous Selective Survival scenario with moderate input parameters; HET2: Heterogeneous Selective Survival scenario with large input parameters.

eTable 2. Parameter Inputs for Cognitive Function Model in Equation A.2 for All Simulation Scenarios

$\beta_{01} U_i + \varepsilon_i + (\beta_{10} + \zeta_{1i})t + (\beta_{20} + \zeta_{2i})t^2$) for all simulation scenarios.*

Parameter	No Selective Survival	HOM1	HOM2	HET1	HET2
b_{00}	0.00	0.00	0.00	0.00	0.00
b_{01}	-1.00×10^{-1}	-1.00×10^{-1}	-5.00×10^{-1}	-1.00×10^{-1}	-5.00×10^{-1}
b_{10}	4.75×10^{-2}	4.79×10^{-2}	4.73×10^{-2}	4.78×10^{-2}	4.79×10^{-2}
b_{20}	-2.95×10^{-3}	-3.03×10^{-3}	-3.35×10^{-3}	-3.30×10^{-3}	-3.33×10^{-3}

*No Selective Survival: Scenario without selective survival; HOM1: Homogeneous Selective Survival scenario with moderate input parameters; HOM2: Homogeneous Selective Survival scenario with large input parameters; HET1: Heterogeneous Selective Survival scenario with moderate input parameters; HET2: Heterogeneous Selective Survival scenario with large input parameters.

eTable 3. Mean Dementia Incidence Rate Ratio for Women vs Men in Each 5-year Age Band Across 1000 Simulated Cohorts for All Simulation Scenarios* Compared With the Adult Changes in Thought Study Reported by Tom et al

	Age band (years)					
	[65, 70)	[70, 75)	[75, 80)	[80, 85)	[85, 90)	[90, 95)
ACT Study	0.51 (0.12, 2.14)	0.69 (0.40, 1.20)	0.86 (0.61, 1.21)	0.91 (0.71, 1.16)	1.27 (0.96, 1.69)	1.10 (0.74, 1.63)
No Selective Survival	1.00 (0.92, 1.09)	1.00 (0.92, 1.09)	1.00 (0.94, 1.07)	1.00 (0.94, 1.07)	1.00 (0.91, 1.11)	1.00 (0.82, 1.22)
HOM1	1.00 (0.92, 1.08)	1.00 (0.92, 1.09)	1.00 (0.94, 1.08)	1.00 (0.94, 1.08)	1.00 (0.91, 1.11)	1.01 (0.82, 1.23)
HOM2	1.00 (0.92, 1.08)	1.01 (0.93, 1.10)	1.02 (0.95, 1.09)	1.01 (0.94, 1.09)	1.02 (0.92, 1.12)	1.00 (0.82, 1.23)
HET1	1.00 (0.93, 1.09)	1.08 (1.00, 1.17)	1.16 (1.09, 1.24)	1.16 (1.08, 1.24)	1.15 (1.05, 1.27)	1.17 (0.96, 1.43)
HET2	1.01 (0.93, 1.09)	1.12 (1.03, 1.21)	1.23 (1.15, 1.32)	1.21 (1.13, 1.29)	1.20 (1.08, 1.32)	1.22 (1.00, 1.51)

*ACT: Adult Changes in Thought Study; No Selective Survival: Scenario without selective survival; HOM1: Homogeneous Selective Survival scenario with moderate input parameters; HOM2: Homogeneous Selective Survival scenario with large input parameters; HET1: Heterogeneous Selective Survival scenario with moderate input parameters; HET2: Heterogeneous Selective Survival scenario with large input parameters.

eFigure 1. Mortality Calibration

(a) Average survival probabilities (conditional on survival to age 50) for men and women across 1000 simulated cohorts compared to survival probabilities in the US 1919-1921 birth cohort. (b) Average simulated mortality hazard ratio for women vs. men $\left(e^{average\left(log\left(\widehat{HR}_{women:men}\right)\right)}\right)$ across 1000 simulated cohorts for all simulation scenarios* compared to the US 1919-1921 birth cohort.

(a)

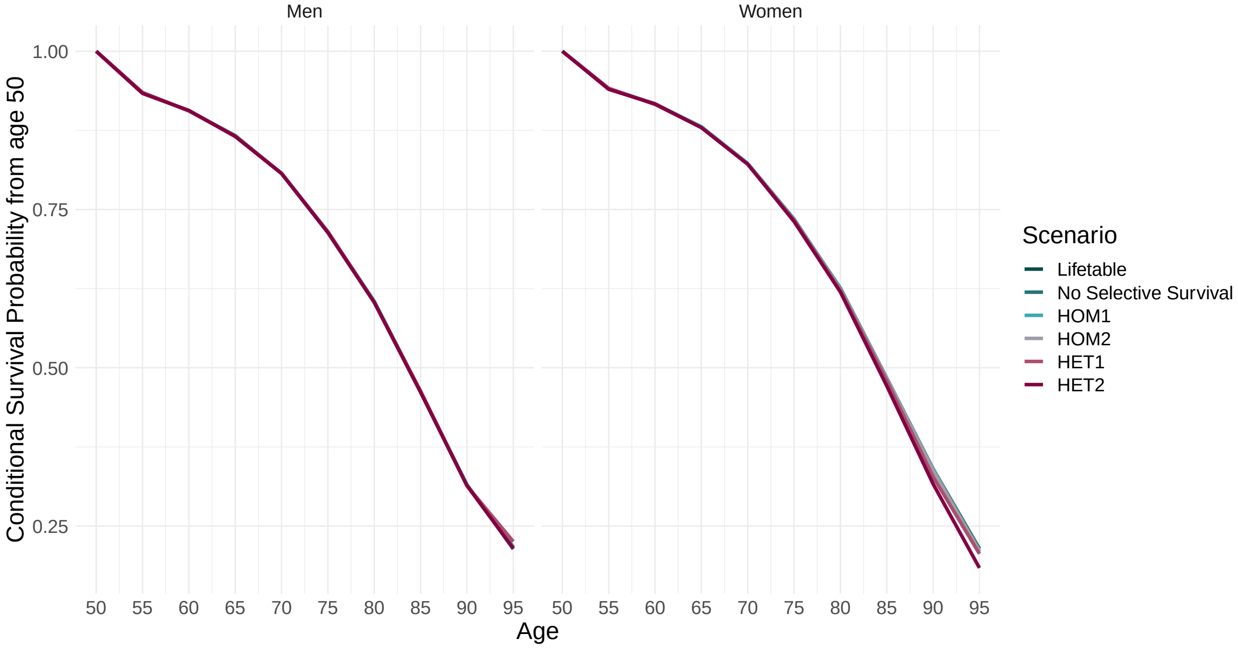

(b)

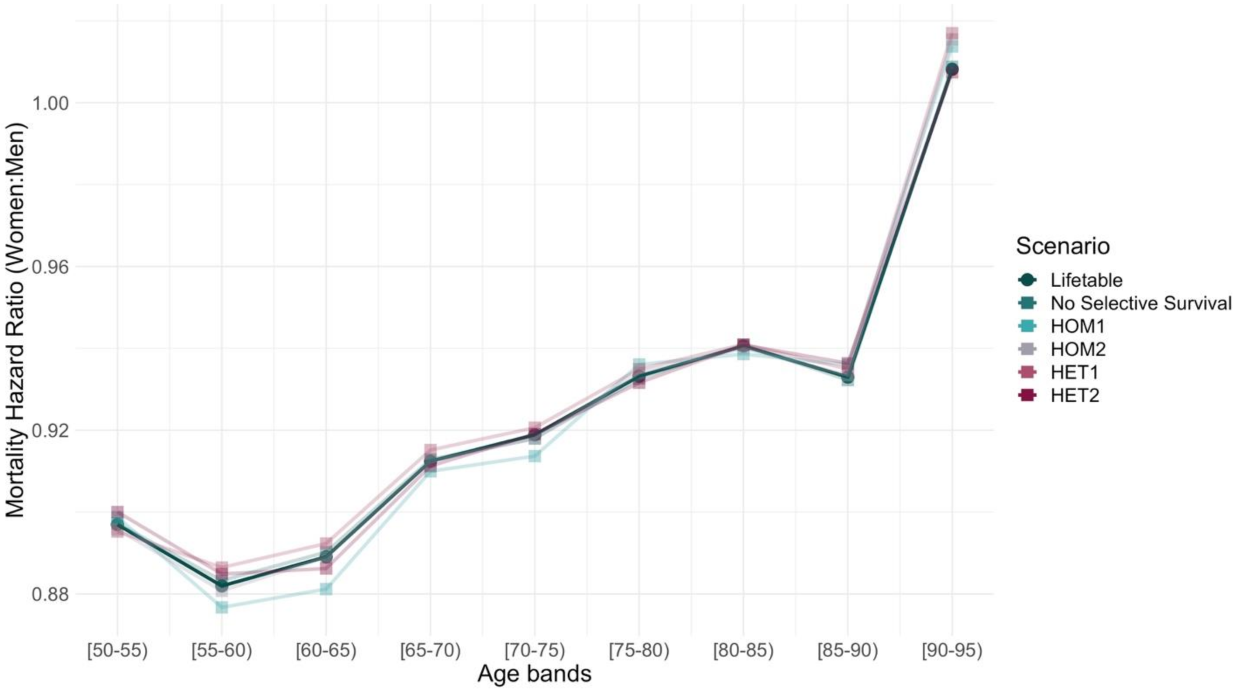

*No Selective Survival: Scenario without selective survival; HOM1: Homogeneous Selective Survival scenario with moderate input parameters; HOM2: Homogeneous Selective Survival scenario with large input parameters; HET1: Heterogeneous Selective Survival scenario with moderate input parameters; HET2: Heterogeneous Selective Survival scenario with large input parameters.

© 2021 Shaw C et al. *JAMA Network Open.*

eFigure 2. Mean Cognitive Trajectories for Surviving Men and Women in Each Simulation Scenario* Superimposed on Cognitive Trajectories for a Random Sample of 100 Individuals

The black horizontal line at -6.5 denotes the age-constant cut point for dementia.

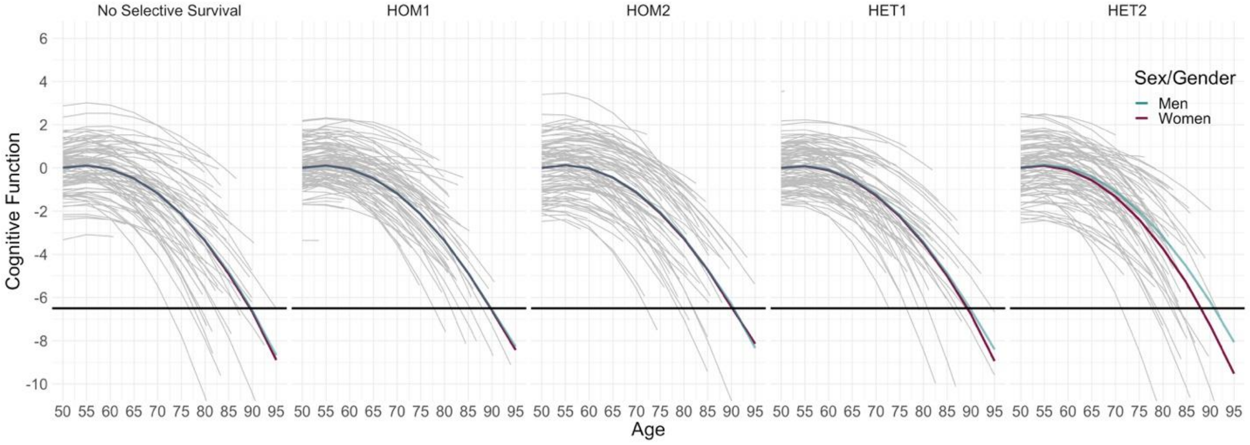

*No Selective Survival: Scenario without selective survival; HOM1: Homogeneous Selective Survival scenario with moderate input parameters; HOM2: Homogeneous Selective Survival scenario with large input parameters; HET1: Heterogeneous Selective Survival scenario with moderate input parameters; HET2: Heterogeneous Selective Survival scenario with large input parameters.

eFigure 3. Dementia Calibration

Average dementia incidence rates for men (used as the reference for calibration) in each simulation scenario* compared to those reported in the ACT study.

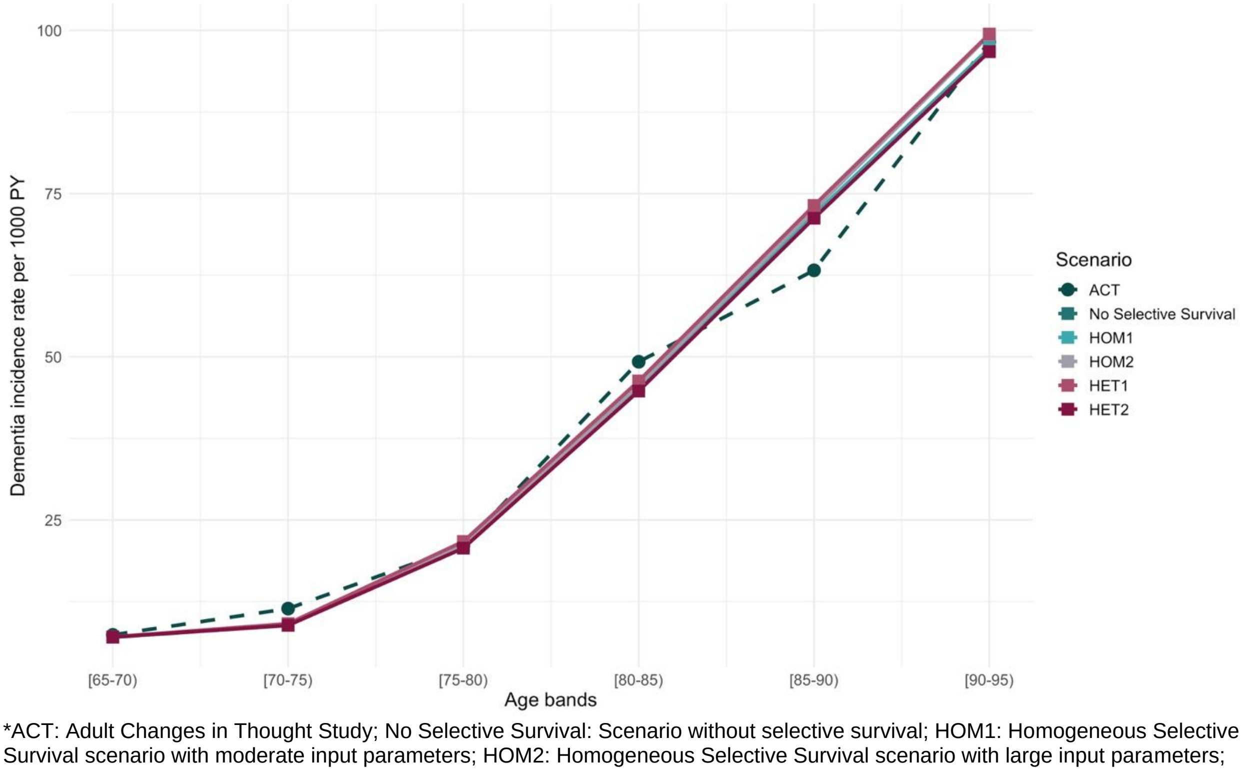

*ACT: Adult Changes in Thought Study; No Selective Survival: Scenario without selective survival; HOM1: Homogeneous Selective Survival scenario with moderate input parameters; HOM2: Homogeneous Selective Survival scenario with large input parameters; HET1: Heterogeneous Selective Survival scenario with moderate input parameters; HET2: Heterogeneous Selective Survival scenario with large input parameters.

© 2021 Shaw C et al. *JAMA Network Open.*
